# Atomically‐Thin Freestanding Racetrack Memory Devices

**DOI:** 10.1002/adma.202505707

**Published:** 2025-06-01

**Authors:** Ke Gu, Prajwal Rigvedi, Peng Wang, Zihan Yin, Hakan Deniz, Andrea Migliorini, Stuart S.P. Parkin

**Affiliations:** ^1^ Max Planck Institute for Microstructure Physics 06120 Halle Germany

**Keywords:** freestanding membranes, local engineering, perpendicular magnetic anisotropy, racetrack memory

## Abstract

Advances in freestanding membranes allow novel heterostructures to be formed from distinct families of materials in 2D or 3D configurations. Recently, this technique has been used to form a 3D racetrack memory device by transferring a complex magnetic thin film heterostructure, in the form of a membrane, onto a corrugated surface. The membrane is released using a water‐soluble oxide layer (Sr_3_Al_2_O_6_). The magnetic structure within the membrane is supported by a thin buffer layer (MgO), which decouples the magnetic structure from the receiving surface. Here it is shown that ultrathin freestanding racetrack membranes can be formed without any buffer layer and that the current‐induced motion of magnetic domain walls within the transferred racetrack is highly efficient. Furthermore, the absence of any buffer layer enables local engineering of the racetracks via their direct coupling with pre‐patterned platinum underlayers on which they are placed. The presence or absence of the Pt underlayer allows for local modulation of the current and field‐induced manipulation of the racetrack magnetization. In addition, the ultrathin freestanding membranes exhibit excellent flexibility and enable highly reliable racetrack devices. The findings highlight the potential of freestanding magnetic heterostructure membranes for advanced spintronic applications.

## Graphical abstract

Ultrathin and flexible freestanding racetrack membranes are fabricated using a water‐soluble Sr_3_Al_2_O_6_ release layer, without a buffer between the release layer and the magnetic heterostructure. The absence of any buffer layer enables local modulation of the current‐ and field‐induced manipulation of the racetrack magnetization via direct coupling with patterned platinum underlayers, showing potential for advanced spintronic applications.

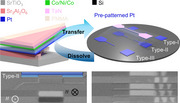

## 1 Introduction

Freestanding membranes, which are typically thin films lifted off from their original substrates, have attracted significant attention because they facilitate the construction of complex heterostructures from dissimilar materials, paving the way for novel functionalities.^[^ ^1^, ^2^, ^3^, ^4^, ^5^, ^6^, ^7^ ^]^ These membranes often exhibit enhanced flexibility due to their reduced third dimension, allowing them to be bent or wrinkled, which makes possible the formation of 3D structures.^[^ ^8^, ^9^, ^10^, ^11^, ^12^, ^13^, ^14^, ^15^ ^]^ To date, research on freestanding membranes has focused primarily on oxides, since oxide thin films can be epitaxially deposited onto sacrificial buffer layers, which are then removed to release the thin film structures.^[^ ^16^, ^17^, ^18^ ^]^ Extending this approach to form freestanding membranes from multilayered metal thin films offers exciting opportunities, particularly in spintronics. One exciting spintronic application is racetrack memory (RTM), in which magnetic racetracks, formed from multilayered metallic thin film heterostructures, encode data as a series of mobile magnetic domain walls (DWs).^[^ ^19^, ^20^ ^]^ This promises high‐density, high‐speed, and energy‐efficient data storage.^[^ ^21^, ^22^ ^]^ It was originally proposed that the most attractive RTM concept would use vertical architectural approaches to maximize the data storage capacity.^[^ ^19^ ^]^ However, the realization is highly challenging using conventional thin film deposition techniques.^[^ ^23^, ^24^, ^25^ ^]^

Recently, we have demonstrated the first 3D RTM devices by utilizing freestanding membranes and taking advantage of their inherent flexibility.^[^ ^26^ ^]^ A stack of thin film materials forming the racetrack was released after dissolving a sacrificial layer of Sr_3_Al_2_O_6_ (SAO) on which they had been deposited. The released membrane was then transferred onto a pre‐patterned surface with micron tall “hillocks” to form 3D racetracks. It was shown that domain walls in these racetracks are highly mobile and could be moved at high speeds by the injection of current pulses. In this previous study, a thin buffer layer of MgO was deposited on top of the SAO layer as it was assumed to be necessary to ensure the uniformity of the ultrathin metal layers sputtered on top. However, this insulating layer prevents the direct interaction between the magnetic membrane and any base layers onto which the metallic membrane is transferred, thereby limiting the range of potential device structures. In this study, we explore the possibility of eliminating the buffer layer so as to form freestanding racetrack membranes with metallic surfaces.

We investigated the influence of the MgO buffer layer by comparing the properties of racetracks formed from atomically‐thin heavy metal/ferromagnetic (HM/FM) heterostructures deposited on various substrates. Our results reveal that SAO itself can surprisingly support the required perpendicular magnetic anisotropy (PMA) of the HM/FM heterostructures, even when they are as thin as ≈4 nm. This is attributed to the small grain size of the Pt layer on SAO. This strong PMA results in rapid DW motion within the racetracks formed from these ultrathin membranes. Subsequent reliability tests confirm the robustness of these membranes. Furthermore, we achieved the local engineering of the racetrack channel through the coupling of the freestanding membranes with pre‐patterned Pt underlayers onto which they are transferred. By injecting pulsed currents in the presence of a small external magnetic field, we can generate DWs at fixed positions, with their separation tunable by adjusting the number of current pulses. The flexibility of the freestanding membranes was demonstrated after transfer onto flexible mica (KMg_3_(AlSi_3_O_10_)F_2_). The present study expands the potential applications of freestanding membranes for RTM devices.^[^ ^27^, ^28^ ^]^

## 2 Results

### 2.1 Deposition of the HM/FM Heterostructures

**Figure 1:**
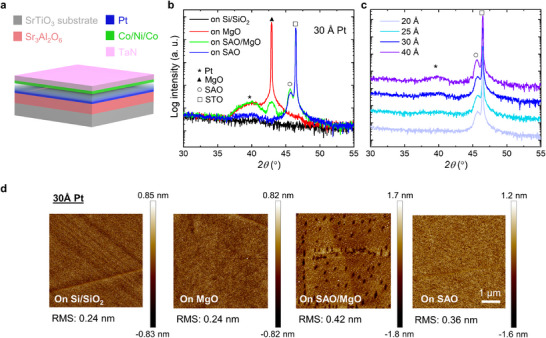
Deposition of HM/FM heterostructures (Pt/Co/Ni/Co) on various substrates with different Pt thicknesses. a) Deposition of SAO/Pt/Co/Ni/Co multilayers on STO (100) substrates and capped with TaN, with Pt thickness variations indicated by the color gradient. b,c) Out‐of‐plane θ‐2θ XRD patterns of HM/FM heterostructures deposited on four types of substrates (b), including Si (100) (covered with ≈250 Å SiO_2_), MgO (100), SAO/MgO, and SAO, and of the heterostructures with Pt thickness varying from 20 to 40 Å deposited on SAO (c). d) AFM images of HM/FM heterostructures deposited on Si/SiO_2_, MgO, SAO/MgO, and SAO. The HM is a 30 Å thick Pt layer.

We deposited HM/FM heterostructures in which the HM layer consists of Pt with thicknesses ranging from 20 to 40 Å and the FM layer is composed of a 3 Å Co|7 Å Ni|3 Å Co trilayer capped with 30 Å TaN. The HM and FM layers were deposited by magnetron sputtering on four distinct substrates: single crystalline Si (covered with ≈250 Å SiO_2_), single crystalline MgO, 200 Å SAO|80 Å MgO bilayer, and 200 Å SAO single‐layer. Hereafter, we refer to these as the as‐deposited samples. A schematic of the sample deposited on the SAO single‐layer is shown in **Figure** **1**a. The SAO and MgO layers were deposited by pulsed laser deposition (PLD) on a SrTiO_3_ (STO) (100) substrate (Figure S1, Supporting Information). The samples with the same Pt thickness were deposited at the same time to ensure consistency (for growth details, see Experimental Section). The X‐ray diffraction (XRD) patterns of the multilayers with different Pt thicknesses deposited on various substrates are compared in Figure 1b and Figure S2 (Supporting Information). A diffraction peak from the face‐centered cubic (111)‐oriented Pt layer can be clearly seen in the samples deposited on MgO and SAO/MgO, while it is relatively weak in the samples deposited on Si/SiO_2_ and SAO. In addition, this peak becomes more pronounced with increasing thickness of the Pt layer (Figure 1c; Figure S2, Supporting Information). Thus, the samples deposited on MgO and SAO/MgO show better crystallinity compared to those deposited on Si/SiO_2_ and SAO, and the crystallinity can be improved by increasing the thickness of Pt. Atomic force microscopy (AFM) imaging shows that all the as‐deposited samples are very smooth, with a root mean square roughness value of less than 0.5 nm (Figure 1d; Figure S3, Supporting Information). The samples deposited on Si/SiO_2_ show the smoothest surfaces, but no clear dependence of the roughness on the Pt thickness can be found.

**Figure 2:**
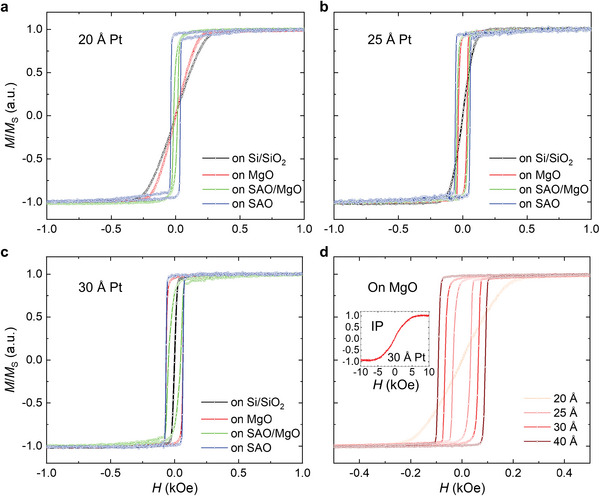
Magnetic properties of HM/FM heterostructures. a,b,c) Out‐of‐plane normalized magnetization (*M*/*M*_s_) versus magnetic field (*H*) curves of as‐deposited HM/FM heterostructures with Pt layer thickness of 20 Å (a), 25 Å (b) and 30 Å (c), respectively. Different colors indicate samples deposited on different substrates: black for Si wafer (covered with SiO_2_), red for MgO substrate, green for SAO/MgO bilayer, and blue for SAO single‐layer. d) Out‐of‐plane *M*/*M*_s_‐*H* curves of as‐deposited HM/FM heterostructures on MgO substrates with Pt thicknesses varying from 20 to 40 Å. The inset shows a typical in‐plane (IP) *M*/*M*_s_‐*H* curve obtained from the as‐deposited sample on MgO with a Pt thickness of 30 Å.

To further evaluate the deposited samples, we performed vibrating‐sample magnetometry (VSM) measurements, as summarized in **Figures** **2** and S4 (Supporting Information). The magnetization‐field (*M*‐*H*) curves of the samples deposited on SAO show the highest squareness, with a sharp switching of the *M* at the coercive field (*H*_c_). Except for the sample with 20 Å Pt, which has a slightly reduced *H*_c_, all the other samples deposited on SAO show almost identical *M*‐*H* curves (Figure S4b, Supporting Information). Moreover, the squareness of the *M*‐*H* curves of the samples improves with increasing thickness of the Pt layer. For example, as shown in Figure 2d, the multilayer deposited on a MgO substrate with 20 Å Pt does not show out‐of‐plane (OOP) magnetic anisotropy, but upon increasing the thickness of the Pt layer, its magnetic easy axis shifts to the OOP direction, and its switching near *H*_c_ becomes more abrupt. Thus, a relatively thick Pt layer is necessary to support the PMA of multilayers, particularly when deposited on Si/SiO_2_ and MgO substrates. In contrast, the SAO layer, serving as an effective buffer, can support the PMA with a very thin Pt layer even down to 20 Å. Furthermore, the surface morphology does not appear to have a significant correlation with the PMA of the samples.

The PMA of Pt (111)/FM heterostructures arises from 3d–5d orbital hybridization at the interfaces (here a Pt/Co interface).^[^ ^29^, ^30^, ^31^ ^]^ This hybridization, combined with Pt's strong spin–orbit coupling, results in anisotropic orbital moments that favor OOP magnetization.^[^ ^32^, ^33^, ^34^ ^]^ The (111) crystallographic orientation enhances these effects due to its favorable symmetry, often leading to a pronounced PMA.^[^ ^35^, ^36^, ^37^ ^]^ However, as discussed above, the origin of PMA in ultrathin HM/FM heterostructures deposited on SAO remains unclear, as indicated by a relatively weak diffraction peak (Figure 1c). To confirm the absence of secondary phases, we first conducted grazing incidence XRD, which revealed no additional peaks (Figure S5, Supporting Information). For further investigations, we transferred the ultrathin membranes (Pt layer thickness: 25 Å) with and without a MgO buffer layer onto Si_3_N_4_ windows after dissolving the sacrificial SAO layer for plan‐view transmission electron microscopy (TEM) analysis. The detailed lift‐off and transfer process is described in the following section and in Experimental Section. The PMA of the transferred membrane was confirmed by Kerr microscopy measurements (Figure S6, Supporting Information). First, we performed selected area electron diffraction (SAED) on the membranes with and without the buffer layer (Figure S7 a,b, Supporting Information). A good crystallinity of the MgO layer was confirmed. Notably, although no pronounced peak of Pt was detected by XRD, the well‐defined rings in the SAED patterns show the polycrystalline nature of the Pt layer. Surprisingly, high‐resolution TEM measurements on these samples revealed that the Pt grain sizes in the sample with the MgO buffer layer (>10 nm) are almost twice as large compared to those in the sample without any buffer layer (typically <5 nm) (Figure S7 c–f, Supporting Information). Thus, the SAO surface promotes the formation of smaller grains. These small grains in membranes without a buffer layer have fewer coherent scattering domains, resulting in diminished interference and, thus, weaker peak intensities in the XRD patterns.^[^ ^38^ ^]^ On the other hand, smaller grain sizes can lead to a more uniform distribution of magnetic properties across the membrane, facilitating the formation of large, switchable magnetic domains and causing abrupt magnetization switching near *H*_c_. Our findings suggest that the nanoscale structure plays a more critical role in determining PMA than the bulk crystallographic texture detectable by XRD.

### 2.2 Racetrack Devices Formed from HM/FM Heterostructures

**Figure 3:**
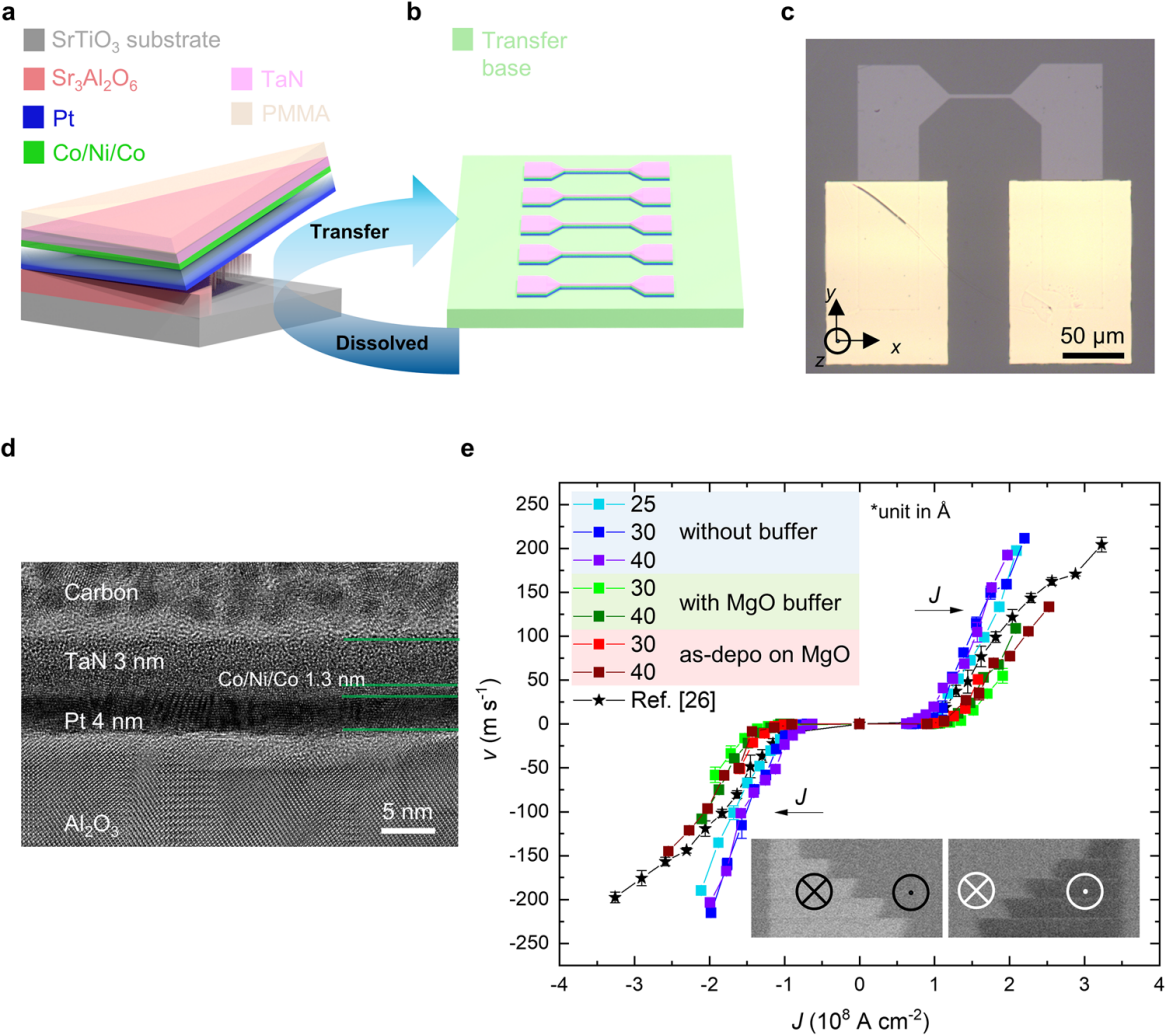
Current‐induced domain wall motion. a) Lift‐off and transfer of freestanding HM/FM heterostructures after dissolving the sacrificial SAO layer. b) Schematic illustration of racetrack memory device fabrication. c) Optical image of a typical freestanding racetrack device, consisting of a straight wire (length: 50 µm; width: 3 µm) and two bonding pads partially coated with Au. The DW moves and currents flow along the *x*‐axis. d) Cross‐sectional high‐resolution TEM image of a freestanding racetrack transferred onto a sapphire substrate with a Pt thickness of 40 Å. Layers from bottom to top: Al_2_O_3_ substrate, 40 Å Pt, 13 Å Co/Ni/Co, 30 Å TaN and carbon capping layer. Green lines indicate interfaces. e) Current‐induced DW velocity in freestanding HM/FM heterostructures (with and without MgO buffer layer) and in as‐deposited HM/FM heterostructures (on MgO substrates) with different Pt thicknesses marked in the figure. Different colors represent different samples: Violet, blue and cyan for freestanding samples without MgO buffer layers; dark and light green for freestanding samples with a MgO buffer; dark red and red for as‐deposited samples on MgO. The black stars represent data from Ref. ^[^ ^26^ ^]^, corresponding to devices with a 50 Å Pt layer and a MgO buffer layer. Link to the Creative Commons License: http://creativecommons.org/licenses/by/4.0/. Insets in e: typical Kerr images of DW motion in response to a series of injected 10‐ns‐long current pulses (*J*  = 1.87 × 10^8^ A cm^−2^) composed of two pulses in freestanding HM/FM heterostructures without a MgO buffer layer and with 25 Å Pt transferred onto a sapphire substrate. The bright and dark regions correspond to down (⊗ or ↓) and up (⊙ or ↑) domains. The error bars in e represent a standard deviation.

After the deposition of the heterostructures on the SAO single‐layer and on the SAO/MgO bilayer, a thin PMMA capping layer was spin‐coated onto the multilayers. These samples were then immersed in deionized water to completely remove the sacrificial SAO buffer layer, enabling the release of the atomically‐thin HM/FM heterostructures. After detaching these heterostructures from their substrates, we transferred them onto sapphire substrates, hereafter referred to as freestanding samples (**Figure** **3**a). Using standard photolithography and Ar ion milling, we then fabricated RTM devices from these as‐deposited (deposited on Si/SiO_2_ and MgO substrates) and freestanding (with or without MgO buffer layer) samples (Figure 3b). A typical RTM device consists of a 50 µm long and 3 µm wide straight wire and two bonding pads, as shown in Figure 3c. To reduce bonding damage to the freestanding samples and to ensure good contact between the freestanding membrane and the bonding wire, we partially covered the two bonding pads with Au. To evaluate our transfer process, we conducted TEM measurements on cross‐sectional lamellae obtained from our freestanding samples (without the MgO buffer layer). Sharp interfaces and clear individual layers are observed, demonstrating a successful deposition and transfer process (Figure 3d). Cross‐sectional high‐resolution TEM images of the membranes formed with various Pt thicknesses are shown in Figure S8 (Supporting Information).

Current‐induced domain wall motion (CIDWM) in these RTM devices was then measured using magneto‐optical Kerr microscopy. The DW velocity of the samples with various Pt thicknesses is plotted against the injected current density, as shown in Figure 3e. We note that our previous experiments have already demonstrated the robustness of the freestanding membrane against the lift‐off and transfer process.^[^ ^26^ ^]^ Therefore, rather than comparing the CIDWM behavior in the samples before and after transfer, we focus on comparing samples deposited on different substrates and with different Pt thicknesses. Interestingly, the freestanding membranes without any MgO buffer layer show the best RTM device performance – highest velocity and lowest threshold current density – compared to the freestanding membranes with a MgO buffer layer and the as‐deposited multilayers on MgO substrates. The PMA supported by SAO is sustained even after transfer, resulting in fast CIDWM in the atomically‐thin RTM devices. Note that the membrane is <7 nm thick including a 3 nm thick TaN capping layer. For direct comparison, we have also included data from our earlier work where the device employed a 50 Å Pt layer and a MgO buffer layer. Notably, while both devices reach a similar maximum domain wall velocity (≈200 m·s⁻¹), the threshold current density required to achieve this velocity is reduced by ≈30% when the MgO layer is eliminated. Furthermore, the CIDWM can be improved by increasing the thickness of the Pt layer. When the Pt layer is relatively thin, the DW motion is relatively slow or cannot be measured. This can be attributed to an increase in defects and pinning sites as the Pt layer thickness is reduced, which is also consistent with the XRD results. By first saturating the magnetization of the sample with a large OOP field (1000 Oe) and then polarizing it with a small field near *H*_c_, these defects can be observed by Kerr microscopy (Figure S9a–d, Supporting Information). These defects favor the formation of small magnetic domains that can be easily switched by currents or fields, thus preventing the generation of a single DW for CIDWM measurements and reducing the maximum current density that can be used to move DWs. With increasing Pt thickness, the density of defects is reduced, facilitating the formation of large magnetic domains and the smooth current‐induced motion of the DWs (Figure S9e,f, Supporting Information). On the other hand, when the squareness of the *M*‐*H* loops is good, i.e., the switching of the magnetization is abrupt, the thickness of the Pt layer does not significantly affect the RTM device performance: a similar CIDWM is found in freestanding racetracks without the MgO buffer, but with Pt layers varying in thickness from 25 Å to 40 Å. These results correspond well with the magnetization measurements and the plan‐view TEM measurements, again demonstrating the versatility of our process and indicating a strong relationship between the squareness of the hysteresis and the CIDWM behavior.

### 2.3 Racetrack Membranes Transferred onto Pre‐Patterned Pt Underlayers

**Figure 4:**
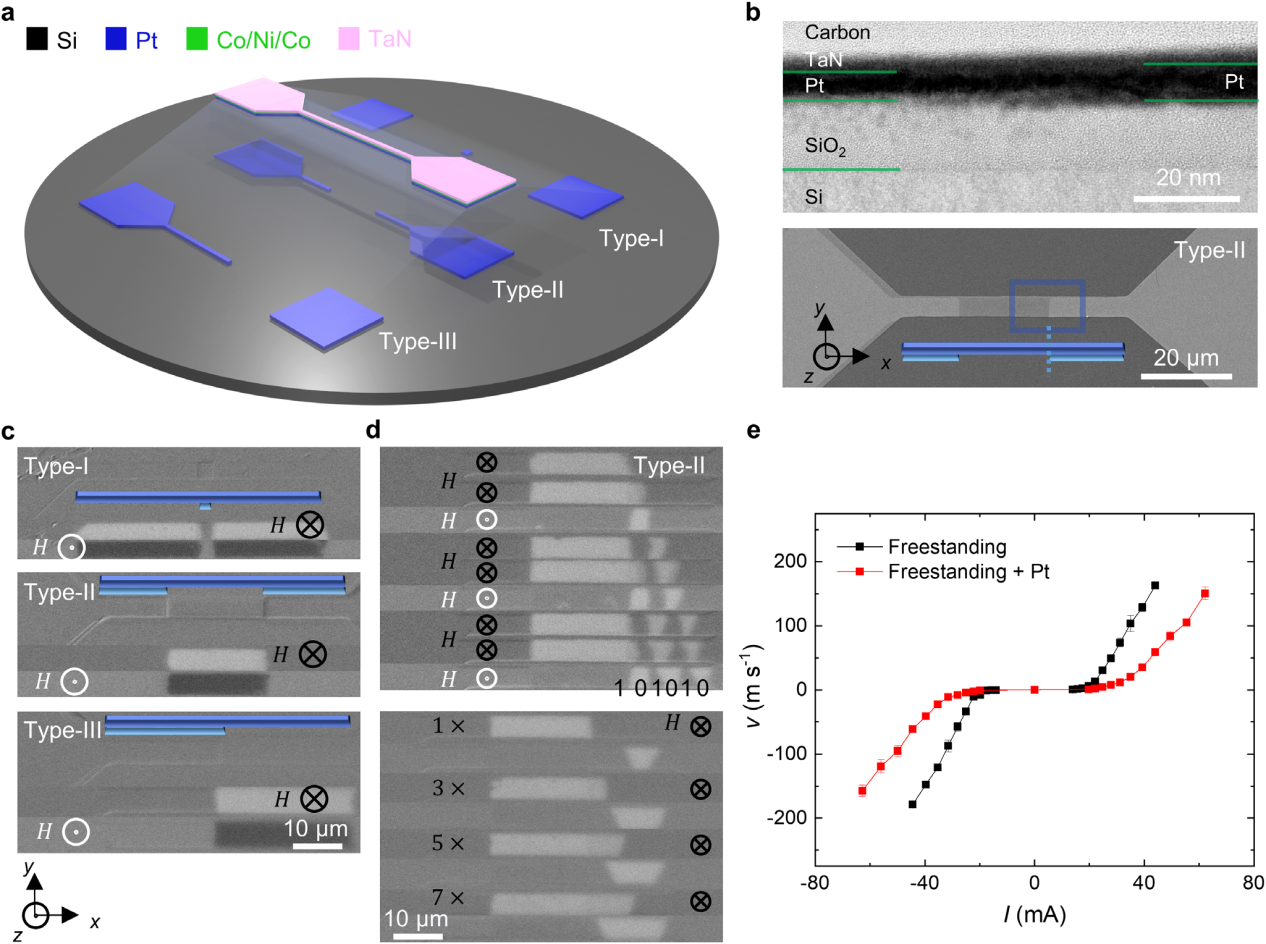
Local engineering of racetrack using pre‐patterned Pt underlayers. a) Schematic illustration of three types of freestanding segmented racetracks with a patterned Pt underlayer formed on a Si/SiO_2_ wafer. From top to bottom: excess Pt positioned in the center (Type‐I), at both ends (Type‐II), and along half of the racetrack channel (Type‐III). b) Top: cross‐sectional TEM image of the transition region in the segmented racetrack, with excess Pt on the right side of the channel. Bottom: SEM image of the top view of the Type‐II segmented racetrack. The blue rectangle in the bottom image highlights the transition region, while the schematic image indicates the thickness of the Pt layer. c) Switching experiments performed in the segmented racetracks. An OOP field of 30 Oe is either pointed up (⊙) or down (⊗) as marked during the switching. Each figure shows one differential Kerr image before switching and two images after switching the magnetization of magnetic domains down or up. A 10‐ns‐long pulsed current is used to generate multi‐domains. From top to bottom: excess Pt is positioned in the center, at both ends, and along half of the racetrack channel (as marked in the schematic); the injected current density in the region without excess Pt *J*_*w*/*o* *Pt*_ is set to 2.52 × 10^8^A cm^−2^, 2.47 × 10^8^A cm^−2^, and 2.52 × 10^8^A cm^−2^, respectively. d) Local generation of DWs in the Type‐II segmented racetrack by alternating the external OOP field (top) and the number of injected current pulses (bottom). Top: each line is taken after injection of a single current pulse (10 ns, *J*_*w*/*o* *Pt*_ = 2.47 × 10^8^ A cm^−2^) with an external OOP field of 30 Oe, directed either upward (⊙) or downward (⊗). A series of domains can be generated as marked by “1” and “0”. Bottom: magnetic domains are switched up and elongated by 1, 3, 5, 7 pulses of currents (10 ns, *J*_*w*/*o* *Pt*_ = 2.47 × 10^8^ A cm^−2^) in the presence of an external field (30 Oe, down ⊗) and then moved out of the ignition region by a series of current pulses (10 ns, *J*_*w*/*o* *Pt*_ = 1.75 × 10^8^ A cm^−2^) composed of ten pulses without any field. Before both of the experiments, the magnetic domains are set to point up (↑) with a large external field (*H*  =  1000 Oe). e) Current‐induced DW velocity in segmented racetracks. Red and black lines represent the velocity in the region with and without the additional Pt layer, respectively. The bright and dark regions in c and d correspond to down (↓) and up (↑) domains. The error bars in e represent the standard deviation. The DW moves and currents follow along the *x*‐axis.

Having demonstrated fast CIDWM in atomically‐thin racetracks based on freestanding multilayer membranes without any MgO buffer layer, we then transferred these membranes onto pre‐patterned underlayers formed from 25 Å Pt on Si/SiO_2_. As shown in **Figure** **4**a, we fabricated three different types of devices, each incorporating an additional Pt underlayer strategically positioned either in the center (Type‐I), at both ends (Type‐II), or along half of the racetrack channel (Type‐III). The transition region between the regions with and without the additional Pt underlayer can clearly be distinguished in scanning electron microscopy (SEM) images. TEM results show that this transition region has no obvious defects (Figure 4b; Figure S10, Supporting Information). This is, in part, due to the improved edge roughnesses of the patterned Pt underlayers that we achieved by post‐polishing after patterning of the Pt layers (Figure S11, Supporting Information).

After fabrication of these “segmented” RTM devices on the patterned Pt underlayer, we first performed switching experiments with injected currents in the presence of an external magnetic field, as shown in Figure 4c. Before applying current pulses, a large external magnetic field of 1000 Oe is used to align any magnetic domains. When a 10‐ns current pulse (*J* ≈ 2.50 × 10^8^ A cm^−2^ in the region without excess Pt, hereafter defined as *J*_*w*/*o* *Pt*_) is injected in the presence of a small opposing field (30 Oe), the region without the additional Pt underlayer can be switched. This occurs because Joule heating depends on the local resistance, and the region without the additional Pt underlayer exhibits higher resistance, resulting in greater heating when current pulses are applied. The increased temperature reduces the coercive field of the FM layer, making it easier for the magnetic domains to be reversed by a small external field. Utilizing this switching mechanism, we can locally create and manipulate DWs. For instance, we alternate the external magnetic field between up and down and use currents to drive the generated DWs to form a series of domains (Figure 4d top; Video S1, Supporting Information). Besides, the size of the domain can also be adjusted by changing the number of injected current pulses (Figure 4d bottom). The magnetic domains are then moved out of the ignition region (region without excess Pt) with small currents (*J*_*w*/*o* *Pt*_ =  1.75 × 10^8^ A cm^−2^). This local control of magnetic domains can facilitate further data processing with DWs.^[^ ^39^, ^40^ ^]^

We then characterized the CIDWM in the segmented racetracks. It is clear that the DW motion becomes slow in the region with excess Pt due to a decrease in the injected current density (Figure 4e; Video S2, Supporting Information). Interestingly, when the DW velocity is plotted against current density instead of current, the curves corresponding to the region with or without additional Pt underlayer almost overlap each other (Figure S12, Supporting Information). Also, the DW can move freely from one region to another across the transition region without any noticeable hindrance. Therefore, we confirm a good adhesion between the Pt underlayer and the freestanding membrane, which is also consistent with our TEM results. In contrast to other reported local modulation techniques–such as ionic liquid gating, subsequent oxidation, and focused ion beam methods^[^ ^41^, ^42^, ^43^, ^44^ ^]^–our approach involving coupling to transferred substrates requires no additional post‐processing after device fabrication. Although we have not yet demonstrated reversible modulation akin to that achieved through gating, the good interface that is formed post‐transfer enables the integration of the atomically‐thin racetrack with other functional materials, such as ferroelectrics and ferromagnets.^[^ ^1^, ^45^ ^]^ This opens up new routes to more complex functionalities, including electrically switchable localized control. Furthermore, our method does not require additional lateral space, as is often the case in 2D geometries,^[^ ^41^, ^46^ ^]^ thereby offering advantages for high‐density device architectures.

### 2.4 Robustness of the Ultrathin Racetrack Membranes

**Figure 5:**
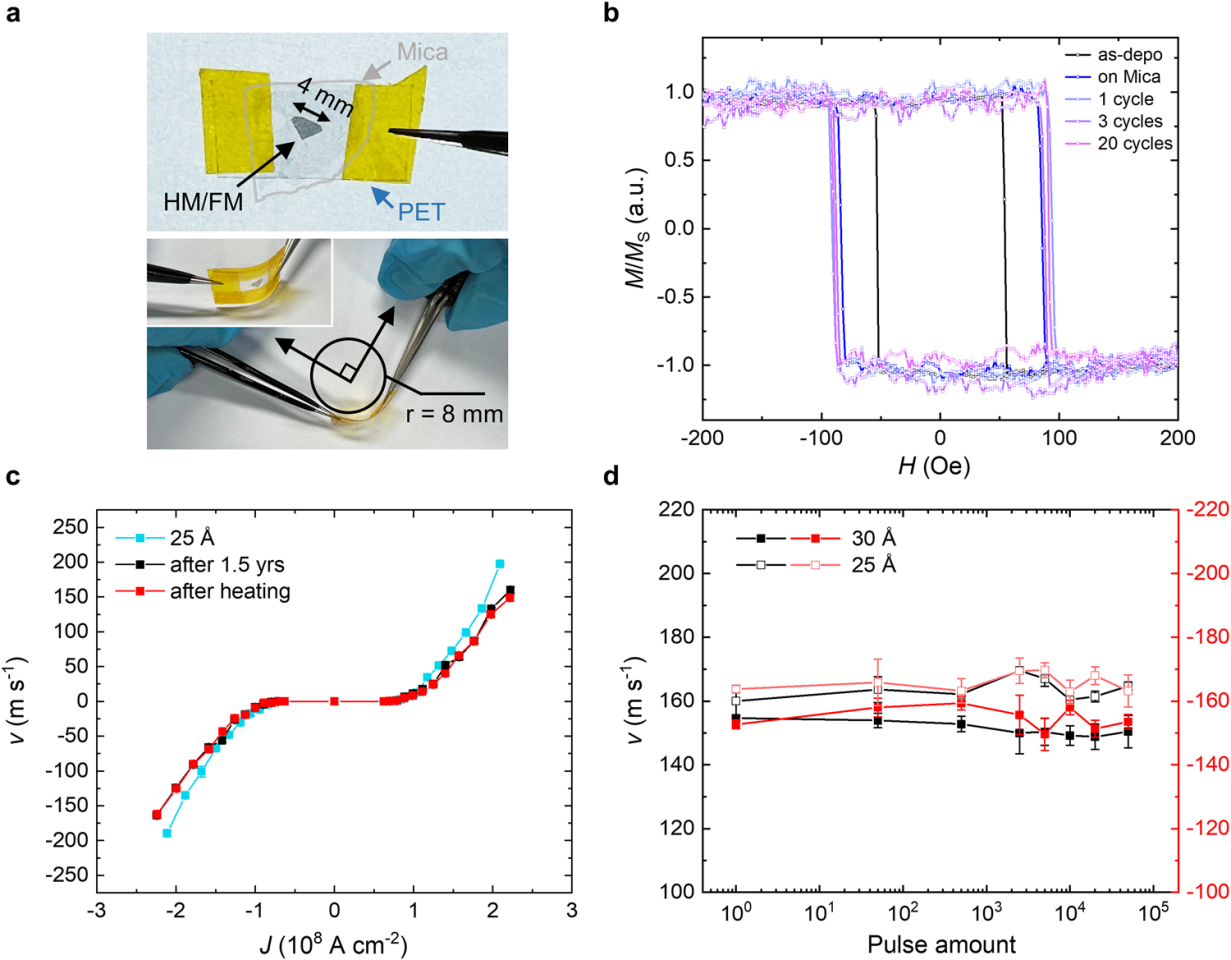
Flexibility and reliability of ultrathin freestanding membranes. a) Bending test setup. Top: freestanding membrane transferred onto a flexible mica substrate, highlighted by grey lines. Bottom: top view of the maximum bending state. The inset in the bottom image shows a side‐view captured during bending. b) Out‐of‐plane normalized magnetization (*M*/*M*_s_) versus magnetic field (*H*) curves measured before, after transfer, and after 20 bending cycles with an 8 mm bending radius. c) Current‐induced DW velocity in ultrathin freestanding heterostructures (25 Å Pt, no MgO buffer). Cyan, black, and red curves correspond to measurements taken immediately after fabrication, after 1.5 years of air exposure, and after 2 h of annealing in air at 100 °C, respectively. d) Current pulse endurance of freestanding membranes without MgO buffer layer, and with either 25 Å (open squares) or 30 Å Pt (filled squares). Black and red lines indicate DW motion along the positive and negative directions, respectively. The error bars in c and d represent a standard deviation. [Correction added on 6 August 2025 after first online publication: Figure 5b has been replaced.]

To further evaluate the robustness of the ultrathin freestanding membranes without any buffer layer, we transferred a freestanding membrane with 25 Å Pt onto a synthetic mica substrate (KMg_3_(AlSi_3_O_10_)F_2_), pre‐cleaved to below 50 µm in thickness to ensure flexibility. The flexible mica, along with the membrane, was then mounted on a polyethylene terephthalate (PET) sheet (**Figure** **5**a top). By mechanically bending the PET sheet, a bending radius of 8 mm was achieved (Figure 5a bottom). Here, a bending cycle is defined as bending to a radius of 8 mm for 10 s followed by release to the original state.

Following the bending cycles, we performed Kerr microscopy measurements on the transferred membrane to characterize its magnetic properties. As shown in Figure 5b, *H*_c_ is slightly increased immediately after transfer, consistent with our previous report.^[^ ^26^ ^]^ Interestingly, both *H*_c_ and the squareness of the *M*‐*H* loops then remain nearly unchanged after up to 20 bending cycles, demonstrating the good flexibility of the ultrathin freestanding membrane. Furthermore, magnetic domains during field switching were observed to grow nearly isotropically (Figure S13, Supporting Information), indicating the absence of noticeable defects such as cracks or grain boundaries induced by bending.

Apart from the flexibility, the air and thermal stability are crucial for the practical integration of the freestanding membranes. To evaluate air stability, we re‐measured the CIDWM in freestanding racetracks (without any MgO buffer layer) after storing the devices in ambient air for 1.5 years (Figure 5c; Figure S14, Supporting Information). Remarkably, almost identical DW velocity versus current density curves were observed, indicating no significant degradation over time. To further assess operational reliability, we conducted current pulse endurance tests by injecting up to 50 000 current pulses (pulses 5 ns long and *J* ≈ 1.91 × 10^8^ A cm^−2^ for the device with 30 Å Pt and pulses of 10 ns in length and *J* ≈ 2.23 × 10^8^ A cm^−2^ for the device with 25 Å Pt). The DW velocity was measured by injecting ≈30 pulses (excluded from the total count) after fixed intervals of 50, 500, 2500, 5000, 10 000, 20 000, and 50 000 pulses. As summarized in Figure 5d, the DW velocity at a fixed current density varies within ± 4% without any obvious trend, demonstrating consistent performance even at high current densities near the operational limit. Following the current pulse endurance tests, we annealed these devices at 100 °C in the air for 2 h, after which the CIDWM behavior was re‐evaluated (Figure 5c; Figure S14, Supporting Information). No apparent degradation was observed, confirming good thermal stability. In short, these results collectively demonstrate the strong robustness of the ultrathin freestanding membranes, underscoring their potential for future spintronic applications.

In conclusion, we have successfully fabricated atomically‐thin freestanding racetracks based on a water membrane‐assisted lift‐off and transfer method. The PMA of the thin HM/FM heterostructure is stabilized on the sacrificial SAO layer, without the need for any buffer layer, and remains intact after its removal, resulting in fast DW motion of up to ≈200 m s^−1^ in the freestanding racetrack. By integrating the freestanding membrane with a pre‐patterned Pt underlayer, we have further demonstrated the local generation of DWs and the manipulation of their velocity. The atomically‐thin freestanding membranes also demonstrate excellent robustness, as confirmed by bending, thermal annealing, air aging, and current pulse endurance tests. Our novel method allows for the integration of atomically‐thin heterostructures with a broad range of exotic materials – such as ferroelectrics, magnetoelectrics, novel superconductors, topological materials, and 2D van der Waals systems – including those formed by distinct techniques or as pre‐patterned structures. With recent advances in sacrificial buffers and amorphous capping layers, crack‐free, high‐quality freestanding membranes can be achieved.^[^ ^47^, ^48^ ^]^ When combined with advanced deposition techniques such as atomic layer deposition or PLD featuring mobile substrate stages,^[^ ^49^, ^50^ ^]^ large‐area membranes with low defect densities become feasible, supporting their practical deployment. Overall, our demonstration of ultrathin freestanding multilayer membranes broadens the application potential of freestanding materials in spintronic devices, paving the way for the development of complex domain wall and skyrmionic devices.^[^ ^27^, ^41^, ^51^ ^]^

## 3 Experimental Section

### Sample Preparation and Device Fabrication

SAO single‐layer and SAO/MgO bilayer films were deposited on STO (100) substrates via PLD using a KrF excimer laser (248 nm). A reflection high‐energy electron diffraction system was employed within the chamber to monitor the growth of the oxide thin films in real‐time. The thicknesses of the SAO and MgO layers were 20 and 8 nm, respectively. During deposition, the O_2_ partial pressure and the laser repetition rate were maintained at 2 × 10^−6^ Torr and 4 Hz for both SAO and MgO. The laser fluence and substrate temperature were set to 0.7 J cm^−2^ and 750 °C for SAO, and 1.7 J cm^−2^ and 700 °C for MgO. Following deposition, the thin films were cooled to room temperature at a maximum rate of 10 °C min^−1^. The samples were then immediately transferred to an ultra‐high vacuum sputtering chamber, using desiccants during the transfer to minimize the effect of humidity. Afterward, multilayers consisting of 20/25/30/40 Å Pt|3 Å Co|7 Å Ni|3 Å Co|30 Å TaN were deposited via magnetron sputtering at room temperature on the four types of substrates, including SAO, SAO/MgO, MgO (100), and 25‐nm‐thick thermally oxidized SiO_2_ from Si (100). For a fixed Pt thickness, four samples on different substrates were sputtered at the same time.

To obtain the freestanding multilayer membrane, a 100 nm thick PMMA layer was initially spin‐coated onto the as‐deposited sample. The entire structure, with or without MgO buffer, was then immersed in de‐ionized water for ≈30 min to fully dissolve the SAO layer. This process allowed the freestanding membrane to detach from the substrate and float on the water surface after a second immersion. The freestanding membrane was subsequently collected and transferred onto various substrates, including Si_3_N_4_ windows, sapphire, Si wafers with pre‐patterned Pt, and flexible mica (KMg_3_(AlSi_3_O_10_)F_2_). The flexible mica substrates were prepared by mechanically exfoliating synthetic mica crystals. A protective PMMA layer was removed in acetone. During the bending tests, the flexible mica substrate was attached to a PET sheet to facilitate the bending process.

Racetrack devices consisting of a 3 µm wide and 50 µm long nanowire and two bonding pads, were fabricated using photolithography and Ar ion milling. The bonding pads were then coated with a 100 nm layer of Au via ion beam sputtering.

To prepare the transfer base with pre‐patterned Pt, the process began with the deposition of 25 Å of Pt on a Si wafer covered with SiO_2_ using magnetron sputtering at room temperature. The desired structures were then fabricated by photolithography followed by Ar ion milling. By gently wiping the surface with lens paper moistened with acetone, an improved edge roughness was achieved (see Figure S11, Supporting Information). Note that, after transferring the freestanding membranes onto the pre‐patterned Pt underlayer, the entire structure was heated up to 150 °C for 10 min to ensure good adhesion of the membrane.

### Film Characterization

The crystal structures of the films deposited on various substrates with different Pt thicknesses were analyzed by high‐resolution XRD using Cu‐Kα_1_ radiation (Bruker, D8 DISCOVER). The surface morphology was characterized by AFM (Bruker, AFM‐Dimension Icon‐PT). Magnetization versus magnetic field (*M*‐*H*) curves were measured by a vibrating‐sample magnetometer (Lake Shore Cryotronics, 8600 Series VSM). Optical imaging of the device was captured using an optical microscope with a 20x objective lens (Zeiss, Axiotron). The SEM image of the device was obtained at a voltage of 5 kV in our dual‐beam microscope (TESCAN, GAIA 3).

### Kerr Microscopy Measurement

*M*‐*H* curves of the freestanding membrane transferred onto flexible mica substrates were measured by Kerr microscopy after the substrate returned to its original flat state. DW velocities were determined from differential Kerr images obtained from the Kerr microscopy measurements using injected current pulses of 10 ns duration (except for pulse endurance tests). In the current pulse endurance tests, pulsed currents with a density sufficient to drive DWs at near‐maximum velocity were injected. An equal number of positive and negative pulses were applied alternately. To prevent multi‐domain formation near edge defects, the DW velocity was measured using 5‐ns‐long current pulses. In the switching experiments, a single current pulse, sufficiently large to generate multiple domains in regions without excess Pt but insufficient to do so in regions with excess Pt, was used to switch the magnetic domain in the presence of an external OOP magnetic field. A magnetic field of 1000 Oe was used to align the magnetization in one direction before each switching experiment.

After air aging and current pulse endurance tests, the devices fabricated from ultrathin freestanding membranes were annealed in air at 100 °C for 2 h. CIDWM measurements were then repeated to evaluate their post‐annealing performance.

All standard deviations come from multiple measurements.

### TEM Investigation

The cross‐sectional lamellae of the atomically‐thin racetrack specimens were prepared by the focused ion beam method (FEI, 600 Nova Nanolab dual‐beam microscope and TESCAN, GAIA 3). Afterward, the lamellae were transferred to TEM grids using a micro transfer system integrated into the dual‐beam system. After fine polishing, the lamellae reached a thickness of a few tens of nanometers. They were then characterized by TEM (JEOL, JEM‐F200) equipped with a Schottky‐type electron source at an accelerating voltage of 200 kV.

For plan‐view TEM investigations, the freestanding membranes were transferred onto TEM grids with Si_3_N_4_ windows, suitable for transmission measurements. Electron diffraction was performed on selected areas of ≈1 µm^2^ in both samples.

## Conflict of Interest

The authors declare no conflict of interest.

## Author contributions

K.G. and S.S.P.P. conceived and designed the experiments. S.S.P.P. coordinated and supervised the research. K.G. performed PLD deposition, transfer of samples, device fabrication, AFM, VSM, and XRD characterizations, and Kerr microscopy measurements. P.W. and P.R. performed sputtering depositions. A.M. optimized the sputtering conditions. Z.Y. and H.D. performed a TEM investigation. Z.Y. prepared the cross‐sectional samples. K.G. and S.S.P.P. wrote the manuscript. All authors contributed to discussions regarding the research.

## Supporting information



Supporting Information

Supplemental Video 1

Supplemental Video 2

## Data Availability

The data that support the findings of this study are available from the corresponding author upon reasonable request.
