# Supplementary material for: Atomically‐Thin Freestanding Racetrack Memory Devices
Source: Adv Mater. 2025 Jun 1;37(33):2505707. doi: 10.1002/adma.202505707 (PMC12369691; doi:10.1002/adma.202505707)
Supplement: Supplementary file 1 — Supporting Information [file ADMA-37-2505707-s001.pdf]

ADVANCED MATERIALS

Supporting Information

for *Adv. Mater.*, DOI 10.1002/adma.202505707

Atomically-Thin Freestanding Racetrack Memory Devices

Ke Gu, Prajwal Rigvedi, Peng Wang, Zihan Yin, Hakan Deniz, Andrea Migliorini and Stuart S.P. Parkin**

Supplementary Information for

Atomically-thin freestanding racetrack memory devices

Ke Gu[1,*], Prajwal Rigvedi[1], Peng Wang[1], Zihan Yin[1], Hakan Deniz[1], Andrea Migliorini[1], Stuart S. P. Parkin[1,*]

[1]Max Planck Institute for Microstructure Physics, 06120 Halle, Germany

*email: guke@mpi-halle.mpg.de; stuart.parkin@mpi-halle.mpg.de

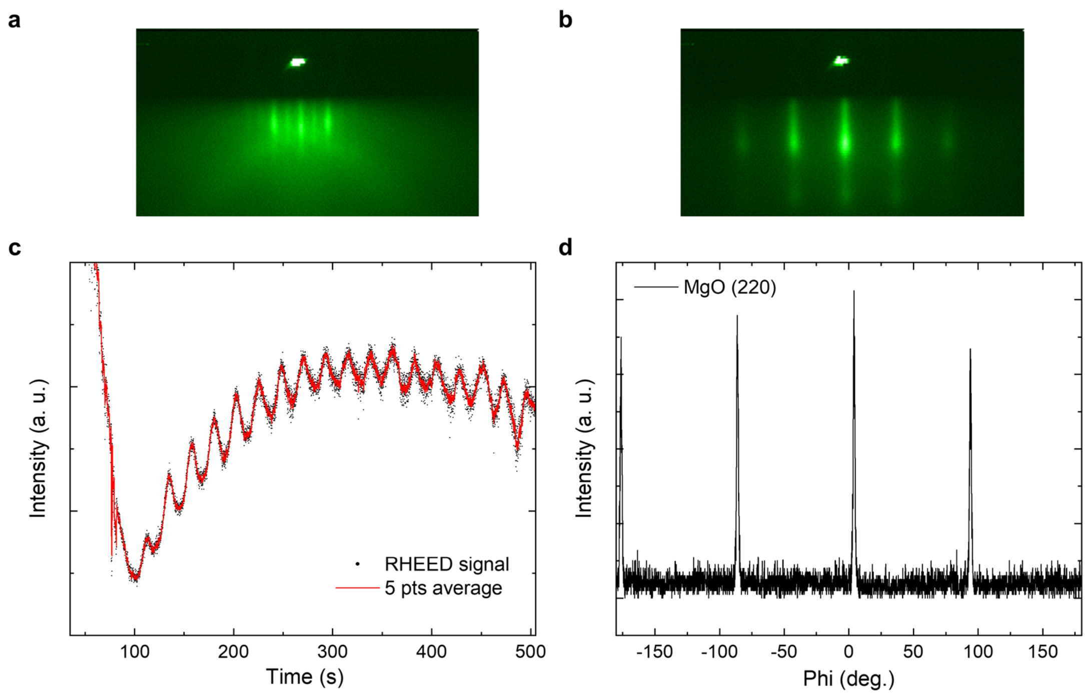

Supplementary Figure S1| Deposition of $Sr_3Al_2O_6$ (SAO)/MgO bilayer thin films. **a**,**b**, In-situ reflection high-energy electron diffraction (RHEED) patterns of SAO (**a**) and SAO/MgO (**b**) deposited on $SrTiO_3$ (100) in a PLD chamber. **c**, RHEED oscillations obtained during the growth of the SAO layer. The red line is the 5-point average of the RHEED signal (black dots). **d,** XRD phi-scan around the MgO (220) peak obtained from a HM/FM heterostructure with an SAO/MgO buffer layer deposited on STO (100). A high surface quality is confirmed by the RHEED patterns taken at the end of the SAO and MgO growth. Layer-by-layer deposition of SAO is shown by the clear oscillations in the RHEED intensity. However, due to the large lattice mismatch between SAO and MgO, layer-by-layer deposition of MgO was not possible. To further confirm the crystallinity of the MgO layer, we performed an XRD phi-scan around the MgO (220) peak. A clear 4-fold symmetry can be observed, indicating the good quality of the deposited MgO layer.

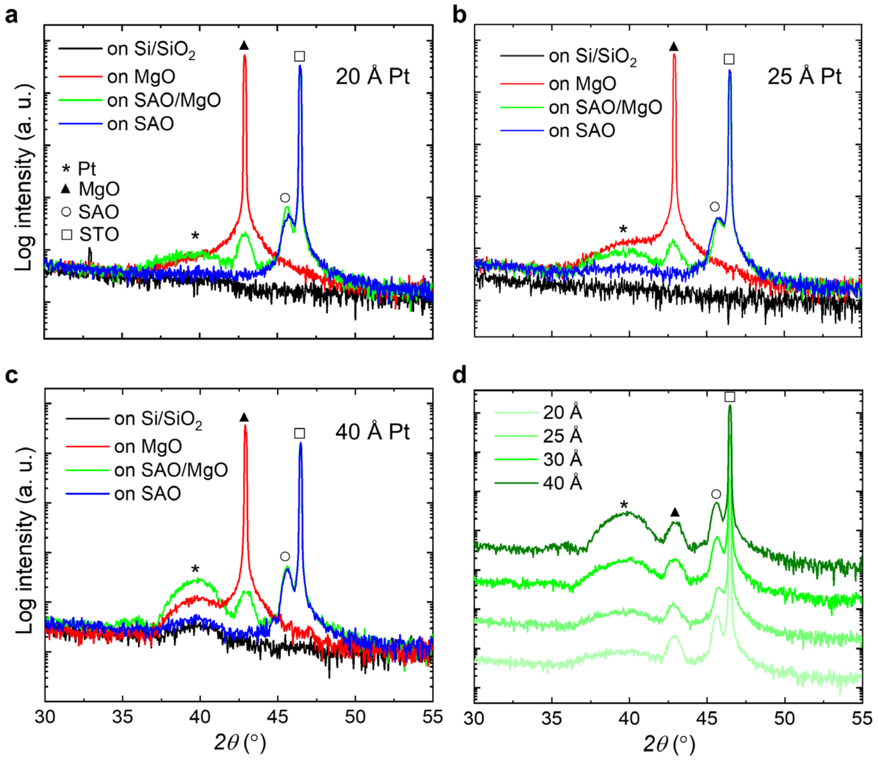

Supplementary Figure S2| Out-of-plane θ-2θ XRD patterns of several HM/FM heterostructures. a,b,c, XRD patterns of samples deposited on four types of substrates (Si/SiO_2, MgO, SAO/MgO and SAO) with different Pt layer thicknesses: 20 Å (**a**), 25 Å (**b**) and 40 Å (**c**). **d,** XRD patterns of samples deposited on SAO/MgO bilayer films with Pt thickness varying from 20 to 40 Å.

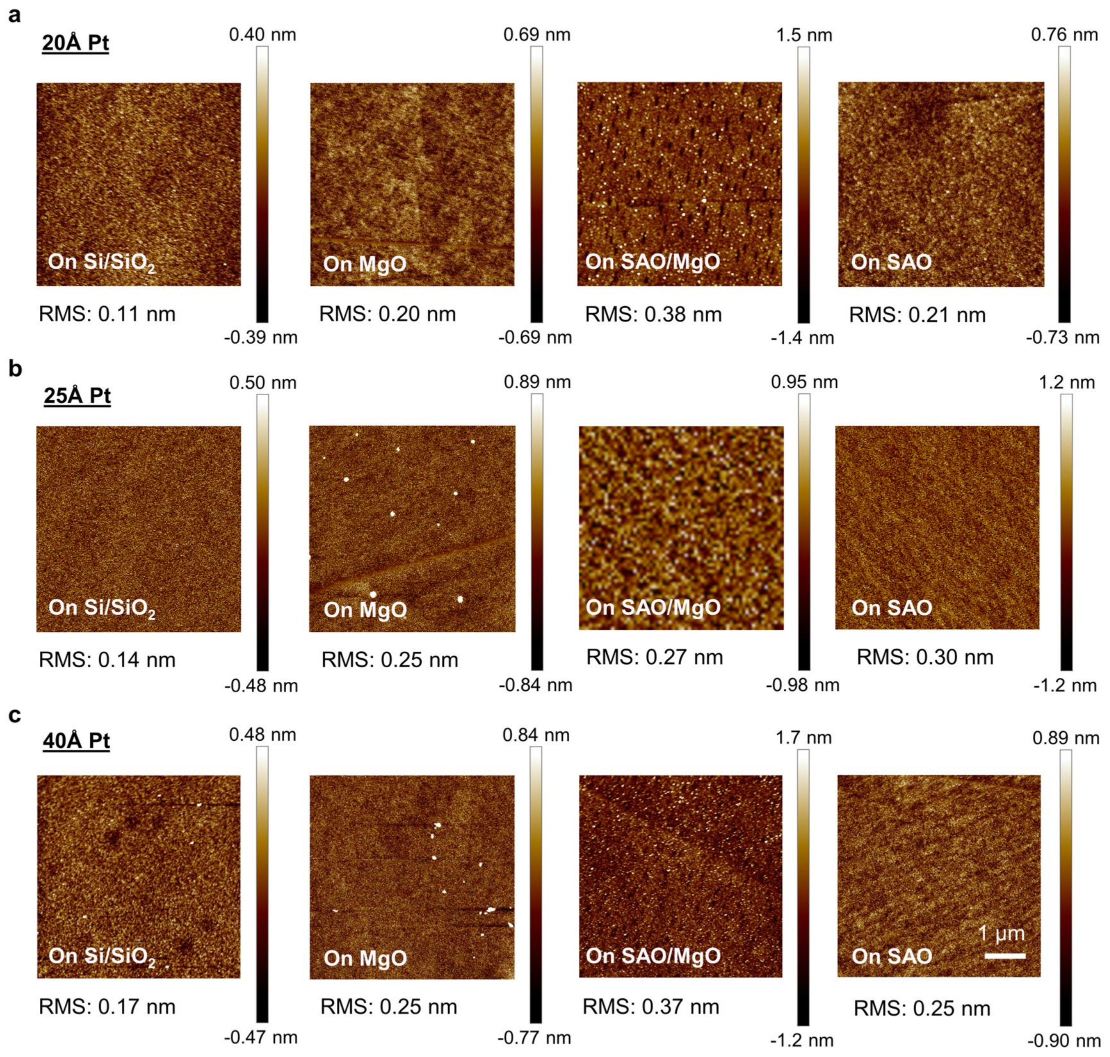

Supplementary Figure S3| AFM images of HM/FM heterostructures deposited on Si/SiO_2, MgO, SAO/MgO, and SAO with a Pt thickness of 20 Å (a), 25 Å (b) and 40 Å (c), respectively.

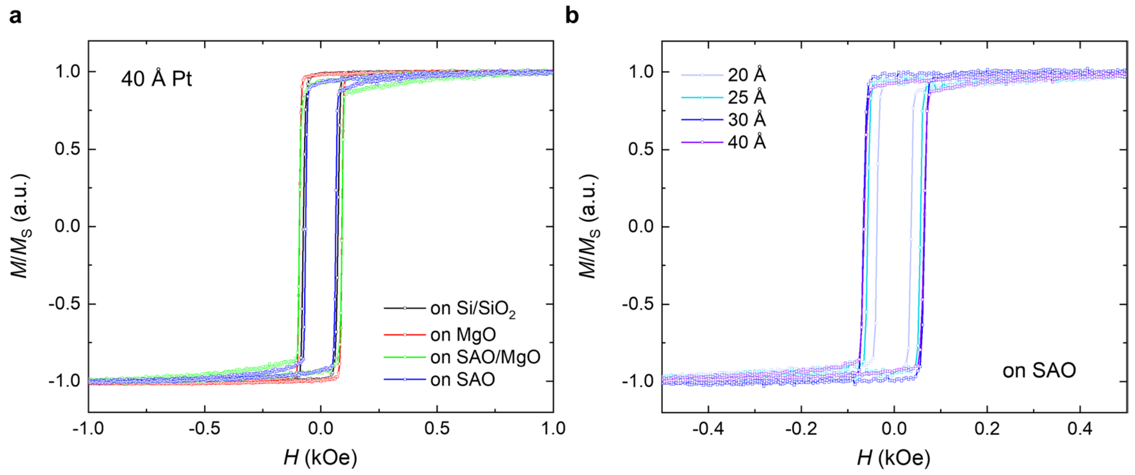

Supplementary Figure S4| Out-of-plane normalized magnetization (M/M_s) versus out-of-plane magnetic field (H) hysteresis curves of as-deposited HM/FM heterostructures with a Pt layer thickness of 40 Å (a) and of as-deposited HM/FM heterostructures deposited on SAO with various Pt thicknesses (b).

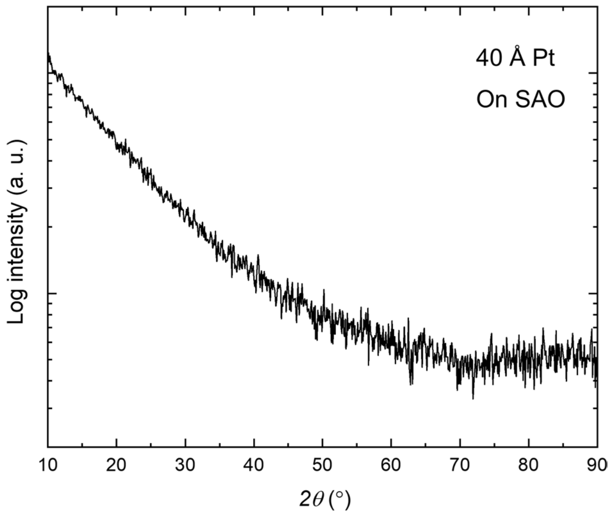

Supplementary Figure S5| Grazing incidence X-ray diffraction pattern of the HM/FM heterostructure deposited on SAO with a Pt layer thickness of 40 Å. The measurement was performed at an incident angle of 1°.

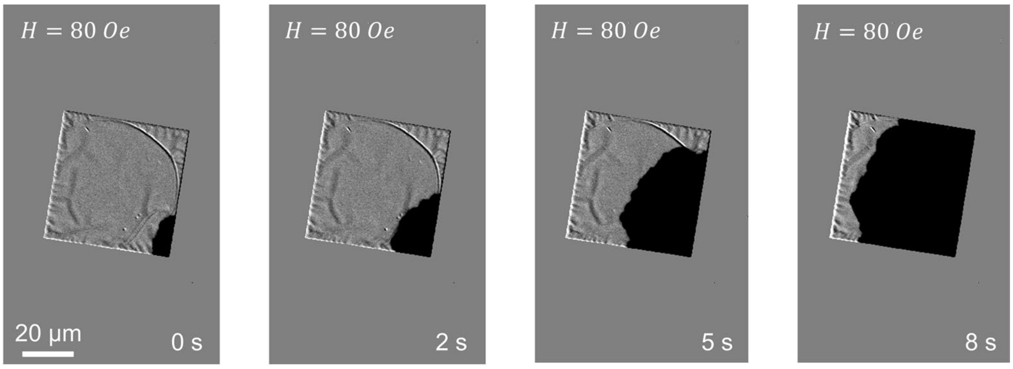

Supplementary Figure S6| Typical Kerr images of the field-induced magnetic domain switching in a membrane transferred onto a Si_3N_4 window. The out-of-plane magnetic field H = 80 Oe. The bright and dark regions within the window correspond to down (↓) and up (↑) magnetization domains, respectively.

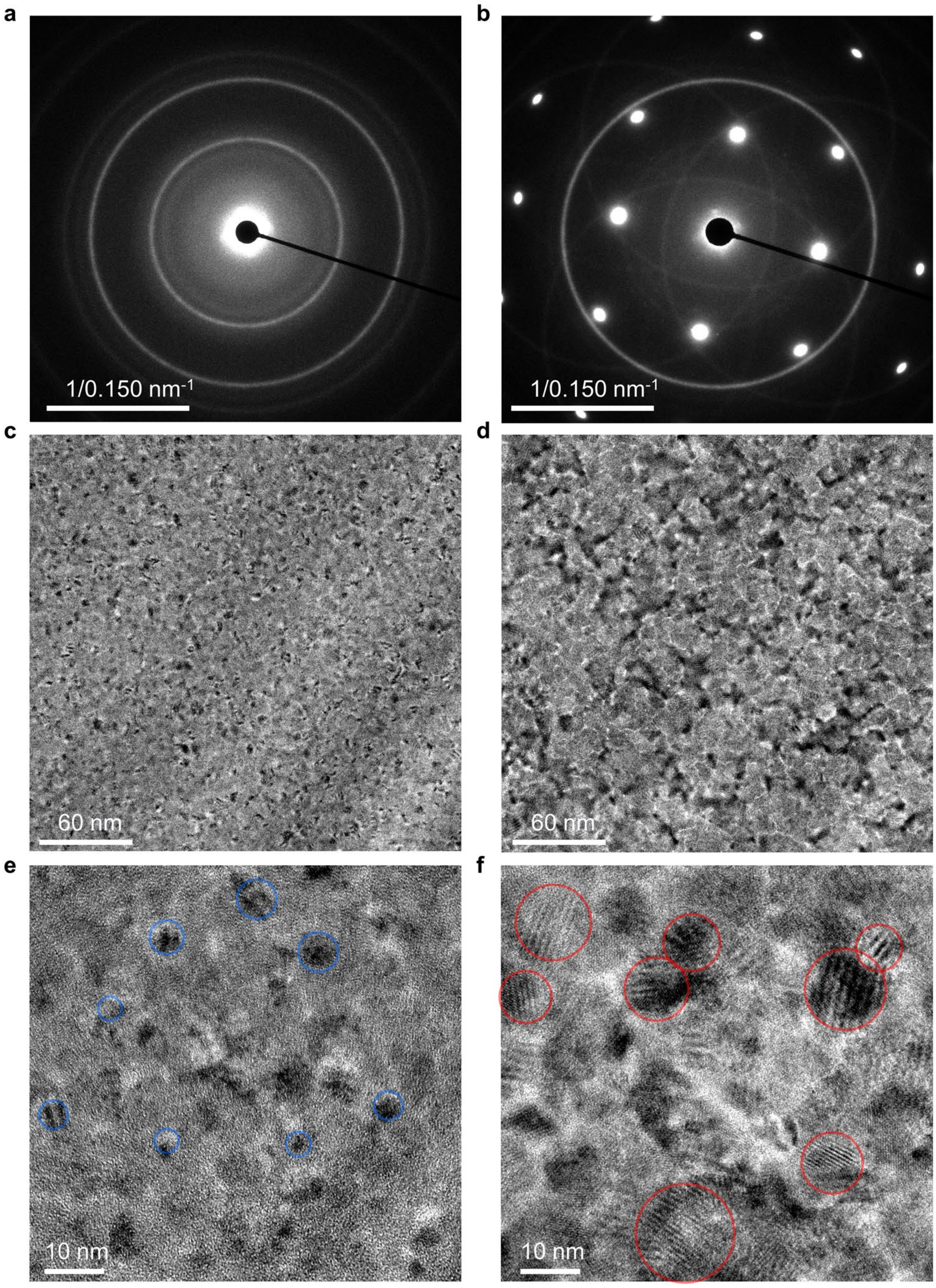

Supplementary Figure S7| Plan-view TEM analysis of freestanding membranes with a 25 Å Pt layer. a,b, Selected area electron diffraction of freestanding membranes without (**a**) and with (**b**) a MgO buffer layer. **c**-**f**, High-resolution TEM images of samples without (**c**,**e**) and with (**d**,**f**) a MgO buffer layer. Typical grain sizes are highlighted with blue circles in **e** and red circles in **f**, illustrating the difference in grain structure.

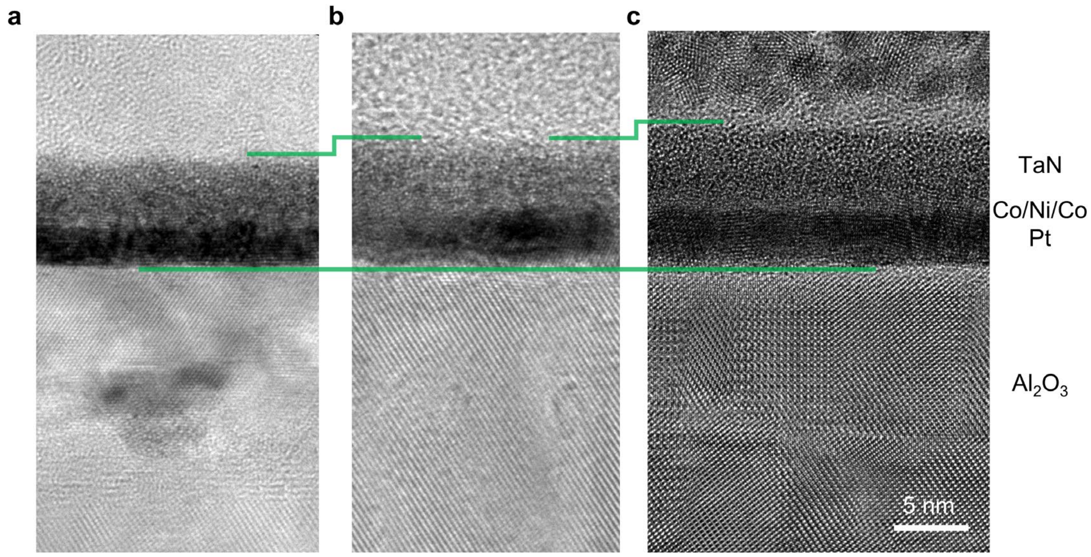

Supplementary Figure S8| High-resolution cross-sectional TEM images of freestanding HM/FM heterostructures transferred onto sapphire substrates, with Pt layer thicknesses of 20 Å (a), 30 Å (b) and 40 Å (c), respectively. Green lines highlight the difference in the total thickness of the stacks.

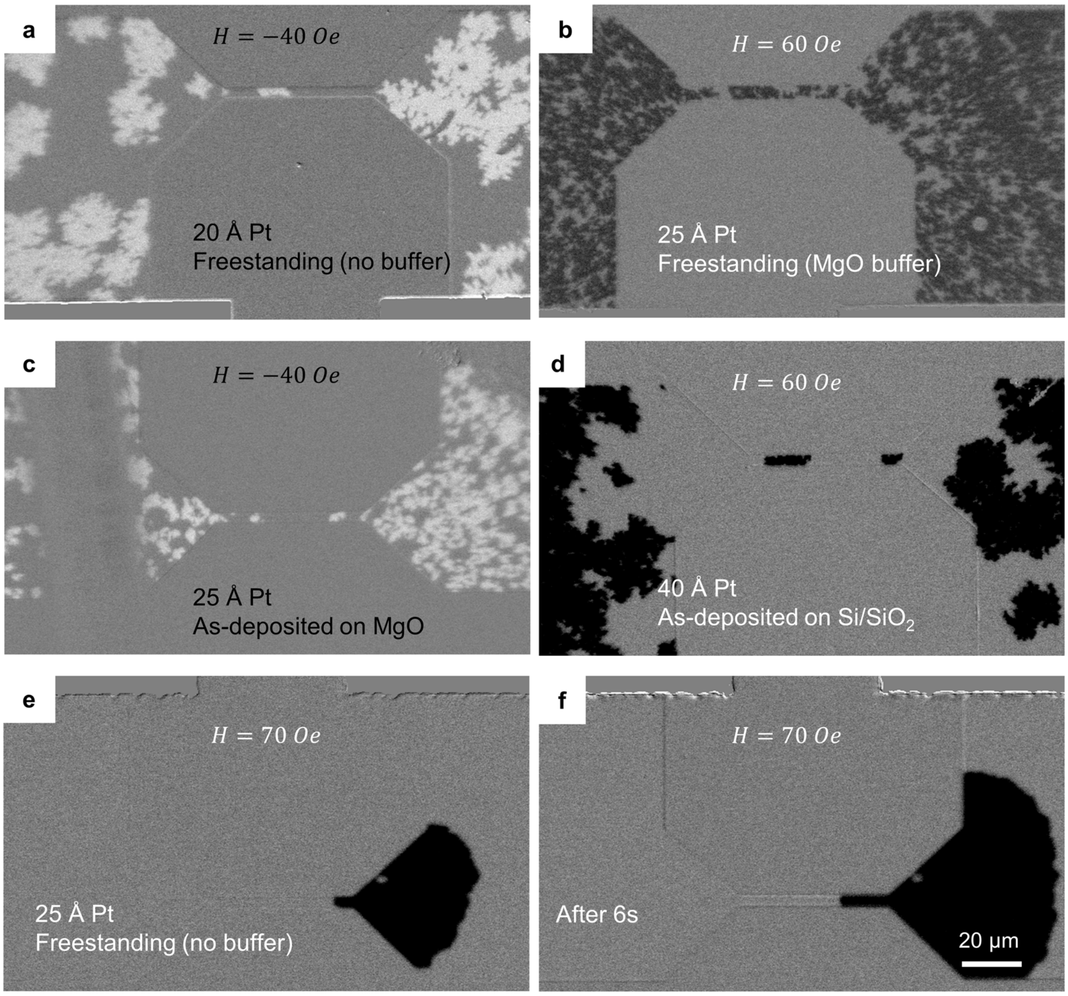

Supplementary Figure S9| Field-induced magnetic domain formation in RTM devices. a-d, Typical Kerr images of multi-domain formation in freestanding HM/FM heterostructures without (**a**) and with (**b**) MgO buffer, and in as-deposited HM/FM heterostructures on MgO (**c**) and Si/SiO_2 (**d**) substrates. **e,f,** Typical Kerr images of a single DW in the racetrack driven by an external field (*H* = 70 Oe). The bright and dark regions correspond to down (↓) and up (↑) domains. The thickness of the Pt layer is marked in each figure.

An external magnetic field close to the coercive field of each sample is applied after a large external field (1000 Oe) has been applied in the opposite direction for a few

seconds. For instance, in the case of Figure **a**, a field of 40 Oe is applied normal inwards after a field of 1000 Oe is applied normal outwards. It is clear that multi-domains are formed after the application of external fields in Figure **a-d**, due to possible defects and pinning sites, some of which are easily switched while others are not. On the other hand, with a thicker Pt layer, a single magnetic domain can be formed after applying the field, as shown in Figure **e** and **f**. In addition, the SAO is the best buffer layer to support the formation of a single magnetic domain among these four substrates (Si/SiO_2, MgO, SAO/MgO and SAO).

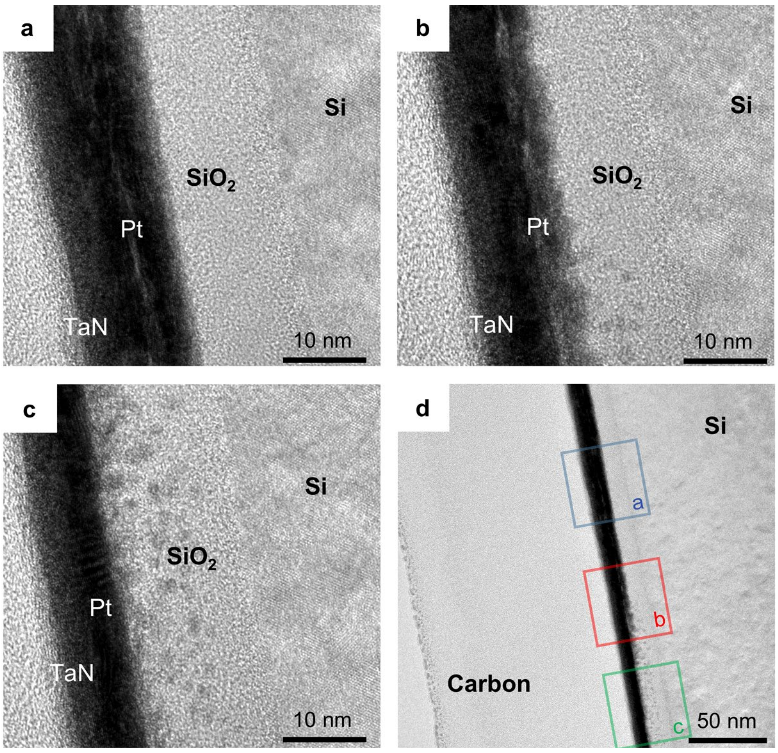

Supplementary Figure S10| Cross-sectional TEM images of the transition region in a segmented racetrack with a pre-patterned Pt underlayer: a, Region with additional Pt layer; **b,** Region with partially etched Pt layer; **c,** Region without additional Pt layer; **d,** Overall cross-sectional image of the transition region. Regions a,b and c are highlighted by blue, red and green rectangles, respectively.

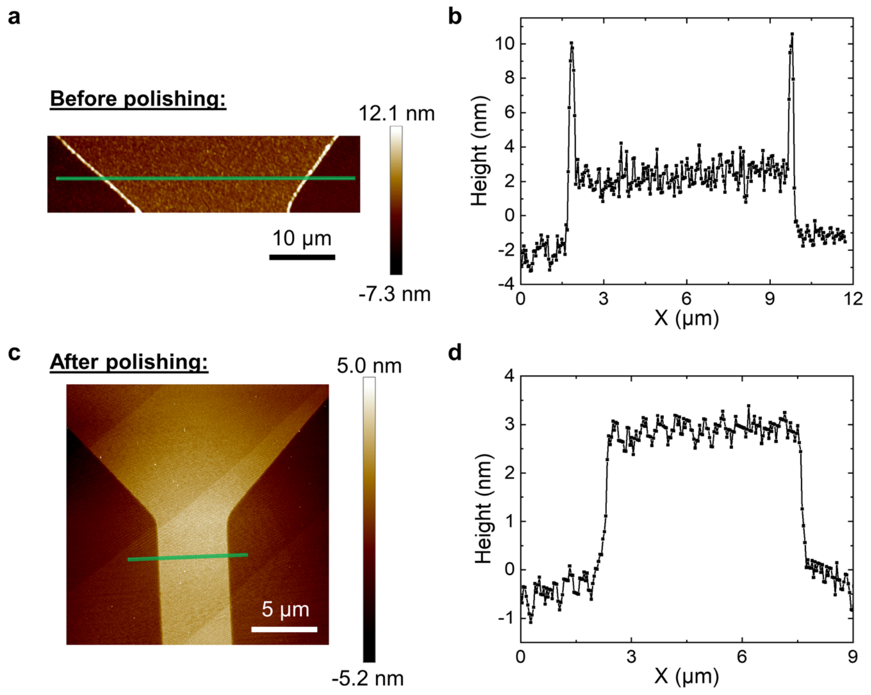

Supplementary Figure S11| AFM images of the pre-patterned Pt underlayer and corresponding line scans of regions marked with green lines. a,c, AFM images of the patterned Pt underlayer before (**a**) and after (**c**) the post-polishing process. **b,d,** Corresponding line scans of the green lines in **a** (**b**) and **c** (**d**), respectively.

The sidewalls formed after photolithography and Ar ion milling can be removed by post-polishing.

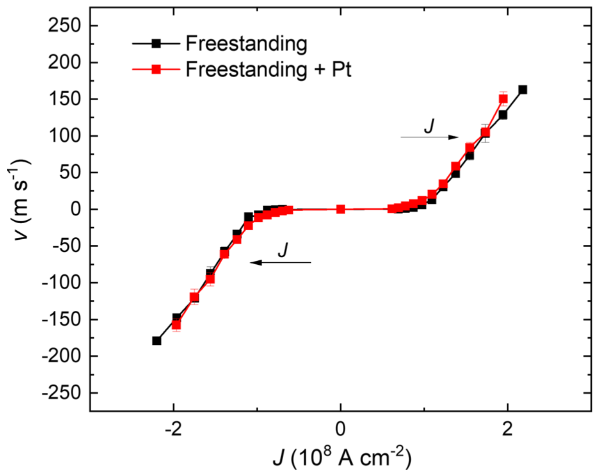

Supplementary Figure S12| Current-induced DW velocity versus current density in a segmented racetrack. The red and black lines represent the velocity in the region with and without the additional Pt underlayer, respectively. The current density in these two regions is calculated from their respective thicknesses. Each error bar corresponds to one standard deviation.

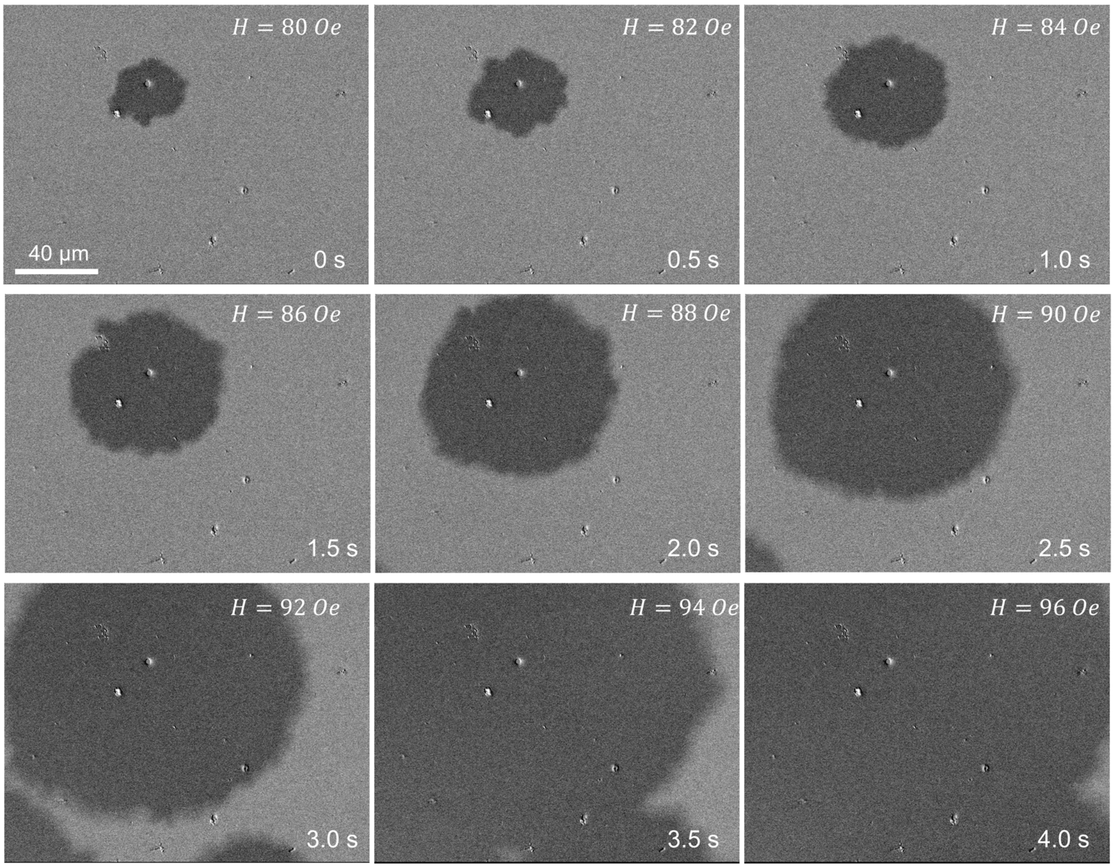

Supplementary Figure S13| Field-induced switching of magnetic domains in an ultrathin freestanding membrane after mechanical bending. Magnetic domain switching observed in a freestanding membrane with a 25 Å Pt layer, after 20 bending cycles with a bending radius of 8 mm. The magnetic domains grow nearly isotropically, indicating that no obvious defects - such as cracks or grain boundaries - are introduced by bending.

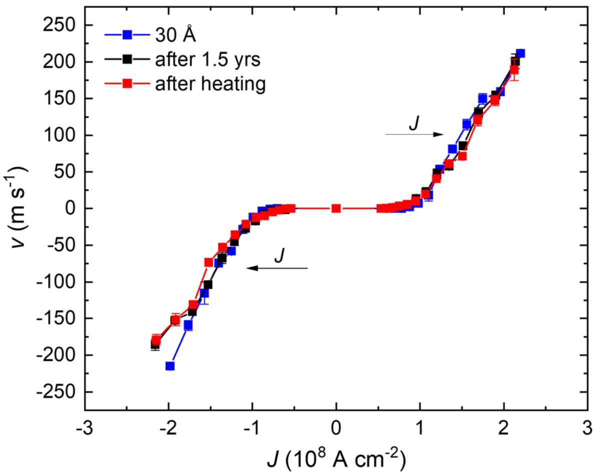

Supplementary Figure S14| Current-induced DW velocity versus current density in racetrack devices after reliability tests. Black and red curves represent data after 1.5 years of air exposure and after thermal annealing at 100 °C for 2 hours in air, respectively. The blue curve corresponds to measurements taken immediately after device fabrication. The devices include no MgO buffer layer and a 30 Å Pt layer. Each error bar corresponds to one standard deviation.

Supplementary Movie caption

Supplementary Movie 1| Kerr microscopy measurements of the continuous generation of magnetic DWs. A single current pulse (10 ns, current density in the region without the Pt underlayer, $J_{w/o\ Pt} = 2.47 \times 10^{8} \mathrm{A\,cm^{-2}}$) is used to heat the ignition region located in the center of the channel (Type-II segmented racetrack) and to drive the DW to the right. An external OOP field of 30 Oe, directed either upwards ($\odot$) or downwards ($\otimes$) as marked in the video, is used to assist the switching of the magnetic domain.

Supplementary Movie 2| Kerr microscopy measurements of DW dynamics in a Type-III segmented racetrack. A series of ten injected current pulses, each 10 ns long ($I = 44$ mA), is used to drive the DW from left to right. The green line marks the transition region between the region with (left) and without (right) the Pt underlayer. For the same current, the DW moves faster in the right half of the channel due to the increase in the current density.
